# Prognostic Indicators for the Early Prediction of Severe Dengue Infection: A Retrospective Study in a University Hospital in Thailand

**DOI:** 10.3390/tropicalmed7080162

**Published:** 2022-07-31

**Authors:** Mayuna Srisuphanunt, Palakorn Puttaruk, Nateelak Kooltheat, Gerd Katzenmeier, Polrat Wilairatana

**Affiliations:** 1 Department of Medical Technology, School of Allied Health Sciences, Walailak University, Nakhon Si Thammarat 80160, Thailand; nateelak.ko@wu.ac.th; 2 Excellent Center for Dengue and Community Public Health, School of Public Health, Walailak University, Nakhon Si Thammarat 80160, Thailand; 3 Hematology and Transfusion Science Research Center, School of Allied Health Sciences, Walailak University, Nakhon Si Thammarat 80160, Thailand; 4 Department of Medical Technology Laboratory, Thammasat University Hospital, Thammasat University, Rangsit Centre, Pathum Thani 12120, Thailand; 5 Akkhraratchakumari Veterinary College, Walailak University, Nakhon Si Thammarat 80160, Thailand; gerd.ka@wu.ac.th; 6 Department of Clinical Tropical Medicine, Faculty of Tropical Medicine, Mahidol University, Bangkok 10400, Thailand

**Keywords:** dengue shock syndrome, dengue hemorrhagic fever, prognostic indicator, multivariate analysis, early prediction

## Abstract

This study aimed to develop simple diagnostic guidelines which would be useful for the early detection of severe dengue infections. Retrospective data of patients with dengue infection were reviewed. Patients with diagnosed dengue infection were categorized in line with the International Statistical Classification of Diseases (ICD-10): A90, dengue fever; A91, dengue hemorrhagic fever; and A910, dengue hemorrhagic fever with shock. A total of 302 dengue-infected patients were enrolled, of which 136 (45%) were male and 166 (55%) were female. Multivariate analysis was conducted to determine independent diagnostic predictors of severe dengue infection and to convert simple diagnostic guidelines into a scoring system for disease severity. Coefficients for significant predictors of disease severity generated by ordinal multivariable logistic regression analysis were transformed into item scores. The derived total scores ranged from 0 to 38.6. The cut-off score for predicting dengue severity was higher than 14, with an area under the receiver operating curve (AUROC) of 0.902. The predicted positive value (PPV) was 68.7% and the negative predictive value (NPV) was 94.1%. Our study demonstrates that several diagnostic parameters can be effectively combined into a simple score sheet with predictive value for the severity evaluation of dengue infection.

## 1. Introduction

Dengue fever is caused by dengue viruses that are transmitted by *Aedes* mosquitoes. The World Health Organization estimates that 2.5 billion people in tropical and subtropical regions worldwide are at risk of infection [1]. Approximately 50–100 million cases are reported annually, of which 500,000 require hospital admission [2]. A significant 71.4% of the cases are children at the age of 2–17 years. The occurrence of dengue fever presents a substantial burden for public health care in developing countries. During epidemiological week 24 of 2022, 1494 dengue cases were reported, leading to a total of 21,689 cumulative reported dengue cases in 2022. This is an increase of 68.7% compared to 12,854 cases reported during the same period (epidemiological week 1 to week 24) in 2021 [3]. Data from Thailand’s surveillance reported 166,680 cases from 2017 to 2022, approximately 27,780 per year. The change of infection was 41.87/100,000 population, with a mortality rate of 0.11% [3].

Currently, there are no drugs for the causative treatment of dengue diseases, and therapies are therefore mainly symptomatic [4]. Nevertheless, the morbidity rate of this disease has been reduced from 5% to about 2% in ten countries in Southeast Asia [4]. Patients with symptoms of a severe infection usually demonstrate hemorrhage followed by shock [5,6]. Common causes of death among children with dengue infection are delayed diagnosis and untimely medical attention, eventually resulting in internal bleeding due to severe hemorrhage and multi-organ failure [7]. The early detection of markers valuable for the diagnosis of disease severity could be imperative for proper medical treatment, preventing the risk of hemorrhagic complications. Laboratory data were shown to support a correct diagnosis of dengue virus infection, thus facilitating treatment and appropriate care plans [8]. Clinical risk factors and laboratory data were recently studied to explore their usefulness for the prediction of dengue disease severity [9,10,11,12]. A decision tree algorithm that differentiates dengue fever from other types of fever was recently proposed [13]. This approach also allowed to forecast the severity of dengue virus infections.

To improve the accuracy of dengue virus fever diagnosis and to simplify the procedures involved, we applied the criteria of the World Health Organization [14,15] and developed a straightforward method for the detection and identification of severe dengue infections. Our model was finally tested by a clinical decision-making approach to validate the accuracy of assessment and diagnosis.

## 2. Materials and Methods

### 2.1. Study Design

This retrospective study was performed by reviewing the previous literature and research to find factors that are associated with the detection and identification of severe dengue infection. Situation analysis and systematic review of clinical risk factors and laboratory tests were also explored [16,17]. The factors were used to create a form for collecting research data and to develop simple methods [17,18] for the detection and identification of severe dengue infections.

### 2.2. Ethics

The study was reviewed and approved by the Human Research Ethics Committee of Thammasat University, Bangkok, Thailand, No. 2 (Document No. 035/2560).

### 2.3. Patient Recruitment and Data Collection

A total of 1185 patients were tested for dengue infection during the years 2017 and 2019 at Thammasat University Hospital in Bangkok, a central urban area of Thailand.

Thammasat Chaloem Phrakiat University Hospital is a public teaching hospital. Its medical center is a tertiary care hospital with 750 beds that provides medical education and training to future and current health professionals. Many teaching hospitals and medical centers are known for their medical research. Close association with medical colleges and universities enhances the research programs at these teaching hospitals.

The sample consisted of 302 patients with a diagnosed dengue infection as revealed by NS1 tests, dengue IgM tests and dengue IgG tests [19]. Patients were classified according to the criteria of the World Health Organization published in 1997 [14], and the classification of severe symptoms used the criteria of the World Health Organization from 2009 [15].

Dates of disease onset as well as patient age were recorded. Patients demonstrating fever at the time of admission were also examined for other underlying diseases.

Data included clinical signs/symptoms and results of laboratory analyses as well as demographic indicators.

Dengue virus serotypes were not routinely diagnosed as the need for specific antisera would result in delayed routine workflow.

Clinical chemistry analysis comprised aspartate transaminase (AST), alanine aminotransferase (ALT), total protein, albumin and the protein/albumin ratio. Immunological assays were used to detect dengue virus nonstructural protein 1 antigen (NS1 Ag), anti-dengue IgM and IgG antibodies.

Immunological/serological tests for dengue IgM, IgG and nonstructural protein 1 (NS1) antigen were performed using the SD BIOLINE Dengue Duo Strip Kit (Standard Diagnostic Inc., Gyeonggi-do, South Korea). Clinical blood chemistry tests for AST, ALT, total protein and albumin were carried out using the Siemens Dimension^®^ RXL Max Clinical Chemistry System (Siemens Healthcare, Ltd., Bangkok, Thailand). Hematological tests for CBC, Hct, Hb and platelets were performed with the DxH 900 hematology analyzer (Beckman Coulter Diagnostics, Switzerland).

### 2.4. Data Analysis

Forecasting risks of various factors to the situation of dengue infection and predictive ability were estimated. Potential predictors for dengue severity, including clinical assessments and laboratory data, were tested for trends with a nonparametric method [9,20]. The predictive ability was analyzed by ordinal binary regression and is presented with coefficients and odds ratios. We included only laboratory tests that are routinely carried out in clinical environments. Results are presented with a 95% confidence interval (CI), odds ratios and dengue risk score. The distribution of scores across the severe and non-severe dengue infection groups is presented by box plots. The classification of infections, including severe and non-severe dengue types, was analyzed by creating a receiver operating characteristic (ROC) graph [21]. A cut-off point for the dengue risk score was used for classification between severe and non-severe types of infection.

## 3. Results

Epidemiological studies have identified indicators for severe dengue infections, which may be useful as prognostic markers for the course of the disease, particularly for pediatric patients [22,23]. We have selected several predictive variables on patient status such as demographic data; clinical manifestations; and hematological, clinical and immunological laboratory results [9].

The total sample population (N) was 1185 people, comprising 635 males (53.6%) and 550 females (46.4%) suspected of being infected with dengue virus. The sample group (n) included 1140 Thai citizens and 45 foreigners. The patients were laboratory-tested at Thammasat University Hospital, Bangkok, Thailand. The prevalence of dengue infection was 28.52% (n = 338), wherein the prevalence for females was 15.53% (n = 184) and that for males was 12.99% (n = 154). A relatively high incidence of dengue infections was found in the age group from 11 to 20 years (26.33%). According to the criteria of the World Health Organization [15], we had a total of 302 patients. When grouped by disease severity, 130 patients had DF (43%), 159 had DHF (52.6%) and 13 had DSS (4.3%). Diagnostic information for these subjects is presented in Supplementary Table S1. Patients were asked whether they had experienced previous dengue infections.

To classify the severity of dengue infection, patients were allocated to three sample groups (Table 1), divided into 107 non-severe infections (35.43%) and 195 severe cases (64.57%). Females (52.8%) demonstrated symptoms of severe dengue fever more frequently than males (47.2%). The mean age of patients with severe infections (26.67 ± 16.55 years) was higher than that of those with non-severe infections (21.81 ± 18.14 years).

Here, the laboratory criteria used to diagnose dengue were NS1, IgM and IgG tests. Formally, we cannot rule out a misclassification of cases as anti-dengue IgG can be detectable for years. It should be noted, however, that we have included only patients who were diagnosed positive by NS1, IgM and IgG. IgG-positive sera may have originated from previous infections that occurred weeks or months ago; however, we did not include IgG results in Table 1.

The parameters are basic or routine parameters in laboratory tests used to assess patients with suspected dengue infection. The six parameters were used to determine the score to predict the severity of infection. This would be helpful in planning treatment and to establish a preliminary guideline for a checklist for laboratory testing. This allows the values from the parameters to be used for dengue risk score calculation, thereby improving accuracy and reducing the service costs of unnecessary laboratory tests. Failure to complete the parameters’ measurement will result in lower risk values.

For the multivariable analysis, the following clinical parameters providing significant predictive ability for infection severity were selected: age (>17 years), anorexia, hematocrit (>40%), neutrophils (≤51%), atypical lymphocyte (>3%), platelet count (≤97 × 10^3^/µL), PT (>13.1 s), PTT (>28.5 s), albumin (≤2.7 g/dL), AST (>104 U/I), ALT (>141 U/I) and immunopositive assays using anti-dengue IgM and anti-dengue IgG (Table 2).

An odds ratio (OR) greater than one indicates a statistically significant probability for a severe dengue infection (*p* < 0.05). The observed rates for six variables (albumin ≤ 2.7 g/dL; AST > 104 U/I; ALT > 141 U/I; platelet count ≤ 97 × 10^3^/µL; PTT > 28.5 s; positive dengue IgM) resulted in risk scores for severe infections of 6.8, 4.8, 9.2, 3.1, 6.8 and 7.9, respectively. The item scores ranged from 0 to 9.22 and the total score ranged from 0 to 38.64.

**Figure 1:**
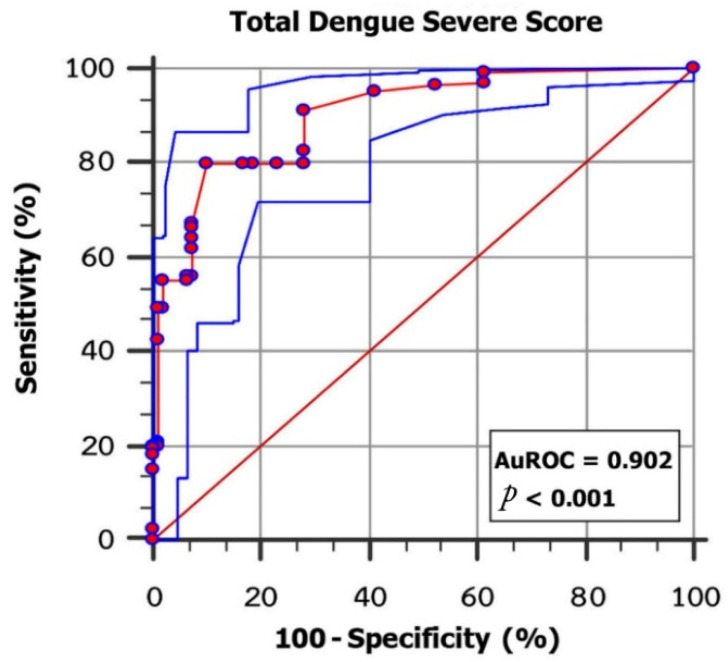
The scoring system for the classification of severe and non-severe infections, with an area under the receiver operation curve (AUROC) of 0.902 (95% CI = 0.863 to 0.933).

**Figure 2:**
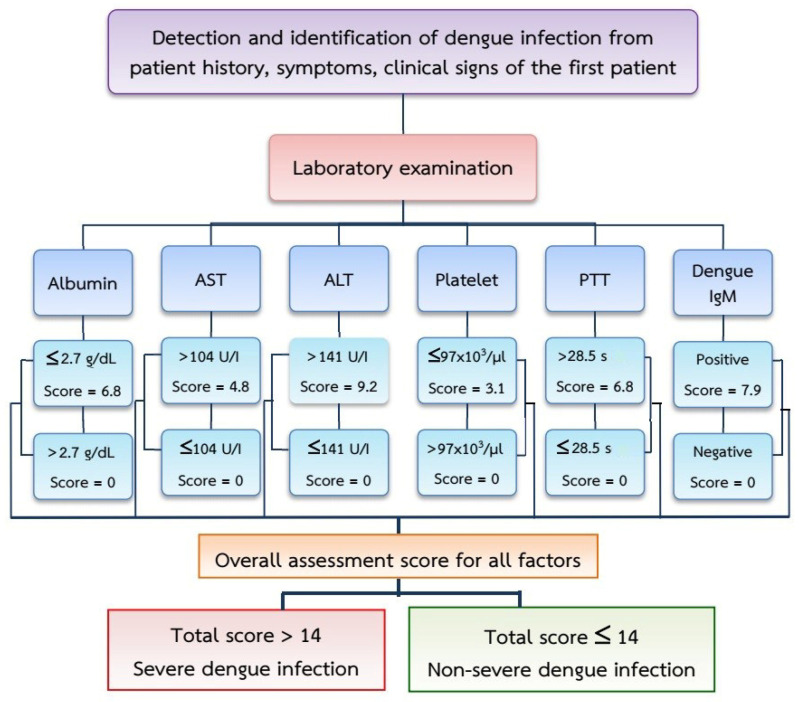
Diagram of procedures for the classification of disease severity with a simple method for detecting and identifying severe dengue infections.

A receiver operating characteristic curve with an area value of 0.902 (95% CI: 0.863–0.933; *p* < 0.001; 80% sensitivity and 89.72% specificity) showed that these six clinical variables provide acceptable discrimination between severe dengue and non-severe dengue infections (Figure 1). Cut-off points were assigned to classify patients in the two severity groups (non-severe and severe), where scores < 14 indicated DF and DHF, while scores > 14 indicated DSS. A graphical presentation of the procedures used for the classification method is presented in Figure 2.

**Figure 3:**
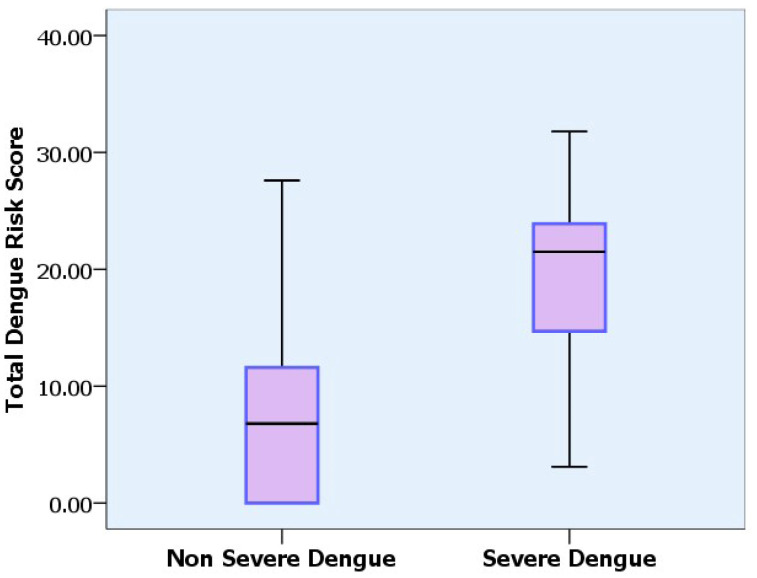
The average dengue risk scores between the groups of non-severe dengue infection and severe dengue infection.

Employing the ROCtab analysis method with total score cut-off points > 14, the model significantly predicts the presence of a severe dengue infection [24]. The prediction by the scoring system was correct with a positive predictive value (PPV) of 68% and a negative predictive value (NPV) of 94.1%. The mean dengue risk scores in patients with non-severe infection and severe infection were 6.6 ± 0.6 and 20.1 ± 0.6, respectively (Figure 3).

The logistic regression analysis of factors for the prediction of severe dengue fever is shown in Table 1. The prediction by the scoring system was correct with a positive predictive value (PPV) of 68% and a negative predictive value (NPV) of 94.1%. In summary, the selected factors that determine predictions for disease progression were proven useful to be incorporated in an uncomplicated diagnostic model for the prognosis of severe dengue diseases.

## 4. Discussion

Analogously to a previously published study, we have designed a straightforward procedure based on clinical data and results from routine laboratory tests that would allow to predict the severity of dengue infection [25]. While previous prospective studies have investigated only dengue infections in children, we have included both children and adults and found an increased risk for greater disease severity in adults by our retrospective study design [26,27]. Compared to earlier reports, we believe that our approach is relatively straightforward and offers advantages in practical terms.

Disease symptoms caused by the dengue virus are generally difficult to diagnose, especially in the early stages of infection [25]. DHF patients may not experience shock symptoms and may not be diagnosed as patients with DSS [4]. Moreover, dengue fever, rickettsia diseases and Q fever can produce similar laboratory data, making it difficult to unambiguously diagnose dengue fever [28]. Epidemiological studies have recently identified several clinical indicators for severe dengue infection [17,29]. A previous study conducted in Thailand found that several clinical parameters can be employed for the prediction of disease severity in pediatric patients [28]. Clinical manifestations may be helpful to detect dengue infections before laboratory results are received [29]. Laboratory tests are sometimes used for confirmation or solely for research purposes and are not used in routine operations [30,31]. Factors usually taken into account for an accurate diagnosis are gender, age, hepatomegaly, stomachache, drowsiness, cold hands and feet, abnormal bleeding, obesity or overweight (in children), malnutrition and swelling of the abdomen. Laboratory data can encompass low counts of white blood cells (<4000/μL), platelet count [32], elevated levels of AST and ALT (SGOT and SGPT) [33], coagulation, increased PTT or PT blood clotting values [34], positive D-dimer tests [29] and congealed bladder wall as observed by ultrasound [35].

Problems with the classification of severe cases arise from the fact that not all DHF cases are severe, and not all mild cases are DF [31,36]. Consequently, the WHO criteria were updated in 2009 [15] and were applied by Tanner et al., who used the results of non-routine laboratory tests to identify dengue infections [37].

As the proportion of atypical lymphocytes increases with shock or fever, we have used an atypical lymphocyte assay [21]. Tests for nonstructural protein 1 (NS1), anti-dengue IgM and IgG using a commercially available assay had previously shown that positive results for dengue IgM and IgG tests were significantly linked to severe dengue infection [20].

We have analyzed variables that affect clinical symptoms and laboratory results for severe and non-severe infections. Severe infections were identified by using a cut-off point higher than 14, thus allowing an accurate identification of the severe disease.

Previous findings as well as our clinical parameter analysis support the diagnostic prediction that patients with a dengue risk score greater than 14 have a high probability of plasma leakage. Cut-off points of <50,000/μL for platelet count and <3.5 g/dL for serum albumin are widely used as indicators of plasma leakage. This finding is in accordance with recent findings reporting a significantly higher proportion of hem concentration (>20%) for severe infections compared to non-severe infections [38]. A study on dengue patients hospitalized between 2010 and 2019 showed that the combination of three variables, namely fluid accumulation, elevated AST level and thrombocytopenia, was associated with severe infections [24]. A meta-analysis reported that four laboratory parameters, namely hemoglobin concentration, hypoalbuminemia, elevated AST level and thrombocytopenia, were significantly associated with DSS [39,40].

Our study may not present a general practice guideline for all outpatient settings as some laboratory investigations such as NS1 rapid tests are required. Furthermore, clinical immunology and biochemistry are not always available in every hospital, eventually leading to reimbursement issues with patients’ health insurance. Therefore, we did not include serum ferritin and other inflammatory markers in this study to avoid unnecessary financial burdens to the patients. The cut-off score for dengue severity was higher than 14, with an area under the receiver operating curve (AUROC) of 0.902. This may eventually result in excessive hospitalizations and unnecessary follow-up procedures. We present here a retrospective study design and an inclusion of patients of all ages with severe dengue infections who are at high risk of mortality. However, further research is needed to understand the benefits and disadvantages of the simple prognostic indicators for the early prediction of severe dengue infection in routine practice and clinical research.

The scoring system we present here is generally applicable to most clinical laboratories and could reduce the likelihood that serious cases of dengue diseases escape medical attention unrecognized.

## 5. Conclusions

A risk score for the disease severity of dengue infections was derived from simple clinical manifestations and the results of routine laboratory examinations. This method can be used in daily practice to assist clinicians to identify patients who demonstrate plasma leakage associated with severe dengue hemorrhagic fever. Correct prediction of the severity of dengue infection contributes to faster diagnosis and reduces unnecessary treatments. However, the effectiveness of the method developed in this study may vary when applied to different populations. Nevertheless, this simple method can be used as a guideline for routine practice in the hospital.

## Figures and Tables

**Table 1:** Significance of factors for predicting severe dengue fever infection.

Parameter: (Predictors)	Category	n	Odds Ratio (OR) *	95% Confidence Interval	*p*-Value	Coefficient *	Score
Albumin (g/dl)	≤2.7	43	6.438	2.23–18.56	<0.001	0.683	0
	>2.7	259					6.8
Aspartate aminotransferase (U/I)	>104	181	6.129	3.65–10.28	<0.001	1.000	0
	≤104	121					4.8
Alanine aminotransferase (U/I)	>141	99	5.133	2.74–9.63	<0.001	0.683	9.2
	≤141	203					0
Platelet count (10^3^/µL)	≤97	190	1.882	1.16–3.06	0.010	0.999	3.1
	>97	112					0
Partial Thromboplastin Time (s)	>28.5	113	7.09	4.00–12.59	<0.001	1.883	6.8
	≤28.5	189					0
Dengue IgM	YES	90	2.407	1.43–4.07	0.001	1.146	7.9
	NO	212					0

* Coefficients from multivariable ordinal logistic regression.

**Table 2:** Clinical parameters of subjects by severity of infection (non-severe infection vs. severe infection as categorized by WHO 2009 guidelines).

Parameter		Non-Severe n = 107		Severe n = 195		*p*-Value
Gender						
Male	(n, %)	44	(41.1)	92	(47.2)	0.321
Female	(n, %)	63	(58.9)	103	(52.8)	
Age (Year)	Mean ± SD	21.81 ± 18.14		26.67 ± 16.55		0.019
<5	(n, %)	12	(11.2)	13	(6.7)	0.001
5–10	(n, %)	18	(16.8)	18	(9.2)	
11–20	(n, %)	37	(34.6)	52	(26.7)	
21–30	(n, %)	17	(15.9)	44	(22.6)	
31–40	(n, %)	10	(9.3)	28	(14.4)	
>40	(n, %)	13	(12.1)	40	(20.5)	
Clinical Profile						
Day of Illness	Mean ± SD	3.49 ± 1.56		3.87 ± 1.5		0.038
Rash	(n, %)	23	(21.50)	42	(21.5)	0.993
Headache	(n, %)	17	(15.90)	34	(17.4)	0.732
Cough	(n, %)	3	(2.80)	3	(1.5)	0.453
Vomiting	(n, %)	27	(25.2)	58	(29.7)	0.400
Abdominal Pain	(n, %)	7	(6.50)	26	(13.3)	0.048
Anorexia	(n, %)	6	(5.60)	29	(14.9)	0.007
Melena	(n, %)	13	(12.10)	27	(13.8)	0.679
Hemodynamic	Mean ± SD					
Systolic Blood Pressure: SBP	(mmHg)	113.84 ± 16.75		113.83 ± 12.64		0.996
Diastolic Blood Pressure: DBP	(mmHg)	89.32 ± 15.47		88.59 ± 14.87		0.689
Pulse Pressure: PP	(mmHg)	24.52 ± 19.17		25.24 ± 19.48		0.758
Hematological	Mean ± SD					
Hematocrit	%	38.19 ± 5.74		41.28 ± 6.24		<0.001
Hemoglobin	g/dL	6.81 ± 7.12		7.51 ± 5.36		0.337
White Blood Cell Count	10^3^/μL	4.39 ± 2.5		5.05 ± 2.88		0.045
Neutrophil	%	48.32 ± 20.9		41.29 ± 17.8		0.004
Lymphocyte	%	36.87 ± 19.17		39.1 ± 14.4		0.295
Atypical Lymphocyte	%	5.38 ± 6.99		11.18 ± 8.58		<0.001
Platelet Count	10^3^/μL	154.65 ± 53.43		47.85 ± 28.39		<0.001
Prothrombin Time: PT	s	13.68 ± 1.76		14.47 ± 3.09		0.050
International Normalized Ratio: INR		1.38 ± 1.69		1.19 ± 0.32		0.246
Partial Thromboplastin Time: PTT	s	36.72 ± 14.92		41.93 ± 22.54		0.016
Biochemical	Mean ± SD					
Total Protein: TP	g/dL	6.77 ± 0.84		6.51 ± 0.86		0.014
Albumin: Alb	g/dL	3.29 ± 0.42		3.14 ± 0.51		0.007
TP/Alb Ratio		2.06 ± 0.19		2.10 ± 0.28		0.110
Aspartate Aminotransferase: AST	U/I	136.48 ± 139.57		405.57 ± 1456		0.058
Alanine Aminotransferase: ALT	U/I	93.7 ± 86.84		222.07 ± 384.68		0.001
Immunological	Positive					
Nonstructural Protein 1: NS1 Ag	(n, %)	71	(66.4)	128	(65.6)	0.901
Dengue IgM	(n, %)	24	(22.4)	66	(33.8)	0.032
Dengue IgG	(n, %)	26	(24.3)	85	(43.6)	0.001

Statistical significance, *p* < 0.05. Mann–Whitney U Test was used to test the difference of mean factors in both groups.

## Data Availability

Not applicable.

## Supplementary Materials

The following supporting information can be downloaded at: https://www.mdpi.com/2414-6366/7/8/162/s1, Table S1: Clinical parameters of subjects by type of infection: dengue fever (DF), dengue hemorrhagic fever (DHF) and dengue hemorrhagic shock syndrome (DSS).

## References

[1] World Health Organization (2009). Dengue and Dengue Haemorrhagic Fever (DHF).

[2] Bhatt S., Gething P.W., Brady O.J., Messina J.P., Farlow A.W., Moyes C.L., Drake J.M., Brownstein J.S., Hoen A.G., Sankoh O. (2013). The global distribution and burden of dengue. Nature.

[3] Department of Disease Control, Ministry of Public Health (2022). Dengue Report.

[4] Carlos C.C., Oishi K., Cinco M.T., Mapua C.A., Inoue S., Cruz D.J., Pancho M.A., Tanig C.Z., Matias R.R., Morita K. (2005). Comparison of clinical features and hematologic abnormalities between dengue fever and dengue hemorrhagic fever among children in the Philippines. Am. J. Trop. Med. Hyg..

[5] Neeraja M., Lakshmi V., Teja V.D., Umabala P., Subbalakshmi M.V. (2006). Serodiagnosis of dengue virus infection in patients presenting to a tertiary care hospital. Indian J. Med. Microbiol..

[6] Dewi L., Nurfitri E. (2012). Pediatric logistic organ dysfunction score as a predictive tool of dengue shock syndrome outcomes. Paediatr. lndones..

[7] Martina B.E., Koraka P., Osterhaus A.D. (2009). Dengue virus pathogenesis: An integrated view. Clin. Microbiol. Rev..

[8] World Health Organization (2006). Report of the Scientific Working Group Meeting on Dengue.

[9] Chang K., Lu P.L., Ko W.C., Tsai J.J., Tsai W.H., Chen C.D., Chen Y.H., Chen T.C., Hsieh H.C., Pan C.Y. (2009). Dengue fever scoring system: New strategy for the early detection of acute dengue virus infection in Taiwan. J. Formos. Med. Assoc..

[10] Suwarto S., Nainggolan L., Sinto R., Effendi B., Ibrahim E., Suryamin M., Sasmono R.T. (2016). Dengue score: A proposed diagnostic predictor for pleural effusion and/or ascites in adults with dengue infection. BMC Infect. Dis..

[11] Marois I., Forfait C., Inizan C., Klement-Frutos E., Valiame A., Aubert D., Gourinat A.C., Laumond S., Barsac E., Grangeon J.P. (2021). Development of a bedside score to predict dengue severity. BMC Infect. Dis..

[12] Liwan A., Gustawan I., Gunawijaya E., Soetjiningsih S., Ariawati K., Hartawan I. (2021). Implementation of Dengue Recurrent Shock Prediction Score in pediatric dengue shock syndrome. Paediatr. Indones..

[13] World Health Organization (1997). Dengue Hemorrhagic Fever: Diagnosis, Treatment, Prevention and Control.

[14] World Health Organization (2009). Dengue Guidelines for Diagnosis, Treatment, Prevention and Control.

[15] Lee I.K., Liu J.W., Chen Y.H., Chen Y.C., Tsai C.Y., Huang S.Y., Lin C.Y., Huang C.H. (2016). Development of a simple clinical risk score for early prediction of severe dengue in adult patients. PLoS ONE.

[16] Soo K.M., Khalid B., Ching S.M., Chee H.Y. (2016). Meta-analysis of dengue severity during infection by different dengue virus serotypes in primary and secondary infections. PLoS ONE.

[17] Pongpan S., Wisitwong A., Tawichasri C., Patumanond J., Namwongprom S. (2013). Development of dengue infection severity score. ISRN Pediatr..

[18] Sangkawibha N., Rojanasuphot S., Ahandrik S., Viriyapongse S., Jatanasen S., Salitul V., Phanthumachinda B., Halstead S.B. (1984). Risk factors in dengue shock syndrome: A prospective epidemiologic study in Rayong, Thailand. I. The 1980 outbreak. Am. J. Epidemiol..

[19] Burke D.S., Nisalak A., Johnson D.E., Scott R.M. (1988). A prospective study of dengue infections in Bangkok. Am. J. Trop. Med. Hyg..

[20] Thein S., Aung M.M., Shwe T.N., Aye M., Zaw A., Aye K., Aye K.M., Aaskov J. (1997). Risk factors in dengue shock syndrome. Am. J. Trop. Med. Hyg..

[21] Streiner D.L., Cairney J. (2007). What’s under the ROC? An introduction to receiver operating characteristics curves. Can. J. Psychiatry.

[22] Thomas L., Brouste Y., Najioullah F., Hochedez P., Hatchuel Y., Moravie V., Kaidomar S., Besnier F., Abel S., Rosine J. (2010). Predictors of severe manifestations in a cohort of adult dengue patients. J. Clin. Virol..

[23] García-Rivera E.J., Rigau-Pérez J.G. (2003). Dengue severity in the elderly in Puerto Rico. Pan. Am. J. Public Health.

[24] Huy N.T., Thao N.T.H., Ha T.T.N., Lan N.T.P., Nga P.T.T., Thuy T.T., Tuan H.M., Nga C.T.P., Tuong V.V., Dat T.V. (2013). Development of clinical decision rules to predict recurrent shock in dengue. Crit. Care.

[25] Cucunawangsih F.K., Dewi B.E., Sungono V., Lugito N.P.H., Sutrisna B., Pohan H.T., Syahrurachman A., Widodo D., Ronoatmodjo S., Sudaryo M.K. (2015). Scoring model to predict dengue infection in the early phase of illness in primary health care centre. Arch. Clin. Microbiol..

[26] Nguyen M.T., Ho T.N., Nguyen V.V.C., Nguyen T.H., Ha M.T., Ta V.T., Nguyen L.D.H., Phan L., Han K.Q., Duong T.H.K. (2017). An evidence-based algorithm for early prognosis of severe dengue in the outpatient setting. Clin. Infect. Dis..

[27] Sachdev A., Pathak D., Gupta N., Simalti A., Gupta D., Gupta S., Chugh P. (2021). Early predictors of mortality in children with severe dengue fever: A prospective study. Pediatr. Infect. Dis. J..

[28] Tantracheewathorn T., Tantracheewathorn S. (2007). Risk factors of dengue shock syndrome in children. J. Med. Assoc. Thail..

[29] Chacko B., Subramanian G. (2008). Clinical, laboratory and radiological parameters in children with dengue fever and predictive factors for dengue shock syndrome. J. Trop. Pediatr..

[30] Deen J.L., Harris E., Wills B., Balmaseda A., Hammond S.N., Rocha C., Dung N.M., Hung N.T., Hien T.T., Farrar J.J. (2006). The WHO dengue classification and case definitions: Time for a reassessment. Lancet.

[31] Bandyopadhyay S., Lum L.C., Kroeger A. (2006). Classifying dengue: A review of the difficulties in using the WHO case classification for dengue haemorrhagic fever. Trop. Med. Int. Health.

[32] Puttaruk P., Srisuphanunt M. (2019). Meta-analysis of the diagnostic accuracy of dengue risk score for dengue virus infection. Thail. Sci. Technol. J..

[33] Halstead S.B., Udomsakdi S., Singharaj P., Nisalak A. (1969). Dengue chikungunya virus infection in man in Thailand, 1962–1964. 3. Clinical, epidemiologic, and virologic observations on disease in non-indigenous white persons. Am. J. Trop. Med. Hyg..

[34] Wichmann O., Lauschke A., Frank C., Shu P.Y., Niedrig M., Huang J.H., Stark K., Jelinek T. (2005). Dengue antibody prevalence in German travelers. Emerg. Infect. Dis..

[35] Shivbalan S., Anandnathan K., Balasubramanian S., Datta M., Amalraj E. (2004). Predictors of spontaneous bleeding in Dengue. Indian J. Pediatr..

[36] Tanner L., Schreiber M., Low J.G., Ong A., Tolfvenstam T., Lai Y.L., Ng L.C., Leo Y.S., Thi Puong L., Vasudevan S.G. (2008). Decision tree algorithms predict the diagnosis and outcome of dengue fever in the early phase of illness. PLoS Negl. Trop. Dis..

[37] Cherry J., Demmler-Harrison G.J., Kaplan S.L., Steinbach W.J., Hotez P.J. (2017). Feigin and Cherry’s Textbook of Pediatric Infectious Diseases.

[38] Nimmannitya S. (1987). Clinical spectrum and management of dengue haemorrhagic fever. Southeast Asian J. Trop. Med. Public Health.

[39] Flamand C., Fritzell C., Prince C., Abboud P., Ardillon V., Carvalho L., Demar M., Boukhari R., Papaix-Puech M., Elenga N. (2017). Epidemiological assessment of the severity of dengue epidemics in French Guiana. PLoS ONE.

[40] Blanchard J., Douglass K., Gidwani S., Khatri U., Gaballa D., Pousson A., Mangla N., Smith J. (2019). Seasonal dengue surge: Providers⬨tm) perceptions about the impact of dengue on patient volume, staffing and use of point of care testing in Indian emergency departments. J. Infect. Public Health.

