# Correction: Srisuphanunt et al. Prognostic Indicators for the Early Prediction of Severe Dengue Infection: A Retrospective Study in a University Hospital in Thailand. *Trop. Med. Infect. Dis.* 2022, *7*, 162

**DOI:** 10.3390/tropicalmed7110339

**Published:** 2022-10-31

**Authors:** Mayuna Srisuphanunt, Palakorn Puttaruk, Nateelak Kooltheat, Gerd Katzenmeier, Polrat Wilairatana

**Affiliations:** 1 Department of Medical Technology, School of Allied Health Sciences, Walailak University, Nakhon Si Thammarat 80160, Thailand; 2 Excellent Center for Dengue and Community Public Health, School of Public Health, Walailak University, Nakhon Si Thammarat 80160, Thailand; 3 Hematology and Transfusion Science Research Center, School of Allied Health Sciences, Walailak University, Nakhon Si Thammarat 80160, Thailand; 4 Department of Medical Technology Laboratory, Thammasat University Hospital, Thammasat University, Rangsit Centre, Pathum Thani 12120, Thailand; 5 Akkhraratchakumari Veterinary College, Walailak University, Nakhon Si Thammarat 80160, Thailand; 6 Department of Clinical Tropical Medicine, Faculty of Tropical Medicine, Mahidol University, Bangkok 10400, Thailand

In the original publication [1], the funder, the New Strategic Research Project (P2P), Walailak University, Thailand, CGS-P2P-2565-069 for Mayuna Srisuphanunt was not included. The authors state that the scientific conclusions are unaffected. This correction was approved by the Academic Editor. The original publication has also been updated.
