# Supplementary material for: Prognostic Indicators for the Early Prediction of Severe Dengue Infection: A Retrospective Study in a University Hospital in Thailand
Source: Trop Med Infect Dis. 2022 Jul 31;7(8):162. doi: 10.3390/tropicalmed7080162 (PMC9416179; doi:10.3390/tropicalmed7080162)
Supplement: Supplementary file 1 [file tropicalmed-07-00162-s001.zip › tropicalmed-1772295-supplementary.pdf]

Tropical Medicine and Infectious Disease

MDPI

Supplementary Material: Prognostic Indicators for the Early Prediction of Severe Dengue Infection: A Retrospective Study in a University Hospital in Thailand

Mayuna Srisuphanunt [1,5,6,*], Palakorn Puttaruk [2,*], Nateelak Kooltheat [1,6], Gerd Katzenmeier [3] and Polrat Wilairatana [4,*]

[1] Department of Medical Technology, School of Allied Health Sciences, Walailak University, Nakhon Si Thammarat 80160, Thailand; mayuna.sr@mail.wu.ac.th (M.S.); nateelak.ko@wu.ac.th (N.K.)

[2] Excellent Center for Dengue and Community Public Health, School of Public Health, Walailak University, Nakhon Si Thammarat 80160, Thailand

[3] Hematology and Transfusion Science Research Center, School of Allied Health Sciences, Walailak University, Nakhon Si Thammarat 80160, Thailand

[4] Department of Medical Technology Laboratory, Thammasat University Hospital, Thammasat University, Rangsit Centre, Pathum Thani 12120, Thailand; palakorn@tu.ac.th (P.P.)

[5] Akkhraratchakumari Veterinary College, Walailak University, Nakhon Si Thammarat 80160, Thailand; gerd.ka@wu.ac.th

[6] Department of Clinical Tropical Medicine, Faculty of Tropical Medicine, Mahidol University, Bangkok 10400, Thailand; polrat.wil@mahidol.ac.th

* Correspondence: mayuna.sr@mail.wu.ac.th (M.S.); palakorn@tu.ac.th (P.P.); polrat.wil@mahidol.ac.th (P.W.)

Table S1. Clinical parameters of subjects by type of infection: dengue fever (DF), dengue hemorrhagic fever(DHF) and dengue hemorrhagic shock syndrome (DSS).

Parameter		DF (n=130)	DHF (n = 159)	DSS (n = 13)	*p*-Value
Gender					
Male	(n,%)	56 (43.08)	73 (45.91)	7 (53.85)	0.722
Female	(n,%)	74 (56.92)	86 (54.09)	6 (46.15)	
Age (Year)	Mean ± SD	23.69 ± 18.13	25.99 ± 16.42	24.85 ± 18.99	0.533
<5	(n,%)	13 (10.00)	11 (6.92)	1 (7.69)	0.312
5-10	(n,%)	19 (14.62)	15 (9.43)	2 (15.38)	
11-20	(n,%)	42 (32.31)	44 (27.67)	3 (23.08)	
21-30	(n,%)	17 (13.08)	41 (25.79)	3 (23.08)	
31-40	(n,%)	18 (13.85)	18 (11.32)	2 (15.38)	
>40	(n,%)	21 (16.15)	30 (18.87)	2 (15.38)	
Clinical Profile					
Day of Illness	Mean ± SD	3.86 ± 1.55	3.67 ± 1.53	3.15 ± 1.21	0.220
Rash	(n,%)	10 (7.69)	53 (33.33)	2 (15.38)	<0.001
Headache	(n,%)	9 (6.92)	38 (23.90)	4 (30.77)	<0.001
Cough	(n,%)	2 (1.54)	4 (2.52)	0 (0.00)	0.733
Vomiting	(n,%)	25 (19.23)	56 (35.22)	4 (30.77)	0.010
Abdominal Pain	(n,%)	1 (0.77)	26 (16.35)	6 (46.15)	<0.001
Anorexia	(n,%)	11 (8.46)	22 (13.84)	2 (15.38)	0.334
Melena	(n,%)	5 (3.85)	31 (19.50)	4 (30.77)	<0.001
Hemodynamic	Mean ± SD				
Systolic Pressure: SBP	(mmHg)	113.62 ± 15.43	113.79 ±13.36	116.54 ± 11.96	0.779
Diastolic Pressure: DBP	(mmHg)	84.86 ± 16.15	91.94 ± 13.13	90.85 ± 17.79	<0.001
Pulse Pressure: PP	(mmHg)	28.76 ± 19.30	21.84 ± 18.83	25.69 ± 20.60	0.010
Hematological	Mean ± SD				
Hematocrit	%	39.05 ± 6.16	41.26 ± 6.07	38.38 ± 6.97	0.006
White Blood Cell count	10^3/µl	4.74 ± 2.60	4.8 ± 2.78	5.72 ± 4.09	0.482
Neutrophils	%	42.73 ± 19.15	44.42 ± 19.46	46.46 ± 17.68	0.666
Lymphocyte	%	40.39 ± 16.74	36.86 ± 15.88	35.15 ± 14.52	0.143
Atypical Lymphocyte	%	8.08 ± 7.99	10.05 ± 9.01	8.38 ± 6.06	0.139

Platelet Count	10^3/μl	102.71 ± 69.69	76.34 ± 57.54	29.85 ± 21.30	<0.001
Prothrombin Time: PT	s.	12.76 ± 1.16	14.64 ± 1.87	18.32 ± 6.45	<0.001
International Normalized Ratio: INR		1.40 ± 2.02	1.20 ± 0.16	1.59 ± 0.76	<0.001
Partial Thromboplastin Time: PTT	s.	28.84 ± 6.37	44.47 ± 16.58	46.98 ± 45.12	<0.001
Biochemical	Mean ± SD				
Total Protein: TP	g/dl	6.64 ± 0.77	6.65 ± 0.88	5.67 ± 0.99	<0.001
Albumin: Alb	g/dl	3.18 ± 0.49	3.25 ± 0.46	2.73 ± 0.53	0.001
TP/Alb ratio		2.12 ± 0.29	2.06 ± 0.22	2.1 ± 0.22	0.164
Aspartate aminotransferase: AST	U/l	173.95 ± 144.53	194.6 ± 180.00	3002.62 ± 5091	<0.001
Alanine aminotransferase: ALT	U/l	127.24 ± 110.66	136.59 ± 124.46	1159.23 ± 1060	<0.001
Immunological	Positive				
Nonstructural Protein1: NS1 Ag	(n,%)	87 (66.9)	102 (64.2)	10 (76.9)	0.615
Dengue IgM	(n,%)	38 (29.2)	45 (28.3)	7 (53.8)	0.152
Dengue IgG	(n,%)	44 (33.8)	62 (39.0)	5 (38.5)	0.662
